# Mechanical Property Tests and Strength Formulas of Basalt Fiber Reinforced Recycled Aggregate Concrete

**DOI:** 10.3390/ma11101851

**Published:** 2018-09-28

**Authors:** Sheng-En Fang, Hua-Shan Hong, Pei-Hui Zhang

**Affiliations:** School of Civil Engineering, Fuzhou University, Fuzhou 350116, China; n160527014@fzu.edu.cn (H.-S.H.); n160520047@fzu.edu.cn (P.-H.Z.)

**Keywords:** fiber-reinforced recycled aggregate concrete, basalt fibers, mechanical properties, strength formulas, optimal volume fraction

## Abstract

In order to investigate the influence of basalt fibers (BFs) on the mechanical performance of recycled aggregate concrete (RAC), some groups of RAC specimens were first tested involving different types of fibers such as carbon fibers, steel fibers, polypropylene fibers and hybrid fibers. The main four indices for the investigation consisted of cube compressive strengths, axial compressive strengths, splitting tensile strengths and Young’s modulus. The effects of fiber volume fractions on the RAC slumps were also discussed. Meanwhile, the mechanical properties and failure modes of the BF-reinforced RAC were compared with those of other fiber-reinforced RAC and common concrete (CC). Subsequently the optimal volume fractions of BFs were explored for different mechanical properties within the volume fraction range of 0–0.2%. The back propagation neural networks were further applied to predict and validate the optimal BF fractions. Lastly, the general strength formulas, as well as the elastic modulus formula, for BF-reinforced RAC were deducted based on the specimen test results. It is found that the addition of fibers may improve the failure modes of RAC and different fibers present positive or negative effects on the mechanical properties. The optimal volume fractions of BF with respect to the four mechanical indices are 0.1%, 0.15%, 0.1% and 0.2% respectively. The proposed strength and elastic modulus formulas of BF-reinforced RAC provide satisfactory predictions with the test results and thus can be used as a reference in practice.

## 1. Introduction

Concrete has been widely used for civil constructions due to its relatively low costs and satisfactory mechanical properties. However, erecting or demolishing concrete structures, as well as concrete production, always produces construction and demolition (C&D) waste that has considerable impact on the environment [1,2,3]. The emissions of carbon dioxide from concrete production are estimated at about 1.35 billion tons annually [1]. Moreover, solid C&D waste also brings problems of land occupation and pollution of groundwater [2,3]. Hence, it has been realized that measures must be taken to deal with such waste.

The utilization of recycled aggregate concrete (RAC) has shown its positive influence on environment and natural resource preservation through reducing the use of non-renewable materials such as natural aggregates [4,5,6,7]. Such utilization also helps the recycling of construction waste. Research has been carried out to explore the different properties of recycled concrete that can be divided into recycled coarse aggregate (RCA) [8,9] and recycled fine aggregate (RFA) [10,11].

However, the mechanical properties of RAC are usually worse than that of common concrete (CC) due to the fact that RCA wrapped in old mortar has some defects that may weaken the joint interface inside RAC [12,13,14]. Some concrete tests also verified this point [15,16,17]. The early research by Nixon [18] reported that the strength reduction of RAC might be up to 20% compared to that of CC. It was also found that the splitting tensile strengths of RAC decreased with the increase of aggregate substitution rates [19]. On the other hand, RFA also presents side effects on the mechanical properties of RAC mainly due to their high water absorption. Khatib confirmed that RAC made with 25% and 100% of RFA had reductions of 15% and 30% in the compressive strengths [20]. Besides that, the other primary side effects of RFA lie in the higher drying shrinkage and less durability of RAC, compared to CC made with natural fine aggregates [21]. These drawbacks highly limit the practical applications of RAC to real-world structures.

It has been realized that fiber-reinforced concrete (FRC) is an effective way to improve the mechanical properties of CC [22,23,24,25]. Fibers can improve the anti-cracking performance of CC by strengthening the interface bonding. With a correct choice of fibers, FRC often presents better compressive and tensile strengths than CC, implying an alternative way for RAC improvement. On the other hand, from a practical point of view, fabricating costs and environmental influence should be taken into account for fiber-reinforced RAC. Therefore, basalt fibers (BFs) made from basalt show their superiority over commonly used fibers such as steel fibers (SFs), carbon fibers (CFs) and glass fibers [26,27,28]. BFs are easier to scatter in concrete than SFs, meanwhile they are significantly cheaper than CFs. BFs process fine thermostability, heat and alkali resistance than other fibers. Most important of all, basalt belongs to the categories of inorganic silicate and thus has satisfactory consistency with cement.

In this study, RCA was used to replace natural coarse aggregate (NCA) for preparation of RAC. RFA were not simultaneously taken into account since the focus on one factor (the replacement of coarse aggregates) could avoid the interactive influence from other factors. The effects of five different fibers of SFs, CFs, polypropylene fibers (PFs), BFs and polypropylene-basalt hybrid fibers (HFs) on fiber-reinforced RAC were compared. Basic mechanical properties such as compressive strengths, splitting tensile strengths and elastic modulus were measured and compared with those of CC. Afterwards, different volume fractions of BFs were also investigated in order to find the optimal fraction and to establish the relationship between different mechanical properties. Meanwhile, back-propagation neural networks (BPNNs) [29] were applied to predict and validate the test results of fiber-reinforced RAC.

## 2. Fiber-Reinforced RAC Tests

### 2.1. Materials

The coarse aggregates for RAC preparation included NCA and RCA. The NCAs came from crushed granite with a grain diameter range of 5–20 mm. The RCAs were made from abandoned concrete with an identical strength grade. The concrete was first artificially crushed before going into a jaw crusher for fine crushing. Then the crushed aggregates were screened and cleaned into different grades. Further physical or chemical treatments were not taken due to the practical consideration that such treatments would highly raise the RAC costs and produce new chemical waste causing environmental pollution. These consequences violate the original intention of using RAC. Table 1 lists the physical properties of both the natural and recycled coarse aggregates.

**Figure 1:**
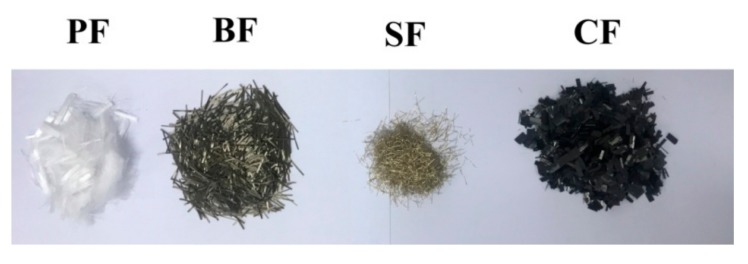
Fibers for recycled aggregate concrete.

The cementitious materials included Portland cement and fly ash. The cement had minimum compressive and rupture strengths of 42.5 MPa and 6.5 MPa, respectively. A Class-F fly ash from a coal-fired power plant was used with a fineness of 11%, a water demand ratio of 94% and a loss of ignition value of 4.0%. The other materials contained river sand, water and superplasticizer. During the agitating process, BFs, CFs, PFs, SFs and HFs (the portion of PF/BF is 1:1 in a volume fraction) were separately added for preparing different kinds of RAC. Table 2 presents the physical and mechanical properties of different types of fibers, as are shown in Figure 1.

### 2.2. Mix Proportions

In order to investigate the actual influence of recycled coarse aggregates on the mechanical properties of RAC, an identical mix proportion was used for both RAC and CC for better comparison in our tests. Therefore, the mix design method for RAC was consistent with CC, which means the recycled coarse aggregates were not pre-saturated. The RAC mix proportions were designed based on the Bolomey formula. The cement was replaced by the fly ash with 10% dosage and the amount of the recycled coarse aggregates remained 50%. The volume fraction for all the fibers were taken as 0.1% for comparison. With respect to the optimal fraction of BFs, the volume fractions were defined as 0%, 0.05%, 0.1%, 0.2%, respectively. The mix proportions for different fiber-reinforced RAC are listed in Table 3 and Table 4.

### 2.3. RAC Fabrication and Test

All the materials were mixed by a forced concrete mixer with a specific sequence. First, all the coarse and fine aggregates were input into the mixer and mixed for 30 s; then the cement and fly ash were added into the mixer and mixed for another 30 s; subsequently the fibers were added and mixed twice (30 s each time) to ensure the uniform dispersion of the fibers. Finally, water and superplasticizer were added together and mixed with the existing materials for 90 s. Segregation or bleeding of concrete was not observed during the agitating process.

The concrete slump was first measured using a slump cone after mixing. After that, the RAC was casted into cube and prism specimens. The cube specimens having a size of 150 mm × 150 mm × 150 mm were used to test the compressive strength ($f_{\mathrm{cu}}$) and the splitting tensile strength ($f_{\mathrm{sp}}$). The prism specimens having a size of 150 mm × 150 mm × 300 mm were used to test the axial compressive strength ($f_{c}$) and the elastic modulus (E). In order to match the practical construction conditions, all the specimens were covered with wet cloth and watered regularly per day. The curing continued for 28 days. Afterwards, they were sent to the laboratory for standard tests [30] using a universal test machine.

## 3. Test Results and Discussions

For each mechanical property index, a group of three specimens were tested and the test results are given in Table 5 and Table 6.

In order to predict and validate the test results, the BPNNs [29] were applied to forecast the mechanical properties of BF-reinforced RAC. Both the training and test samples were derived from the test results. At the same time, the mechanical properties of BF-reinforced RAC with a volume fraction of 0.075%, 0.15% and 0.25% were predicted.

Based on the test results, the conversion formulas of cube compressive strengths to axial compressive strengths, splitting tensile strengths and elastic modulus of RAC were presented respectively. Furthermore, the calculation formulas for basic mechanical properties of BF-reinforced RAC were also established.

### 3.1. Slumps

**Figure 2:**
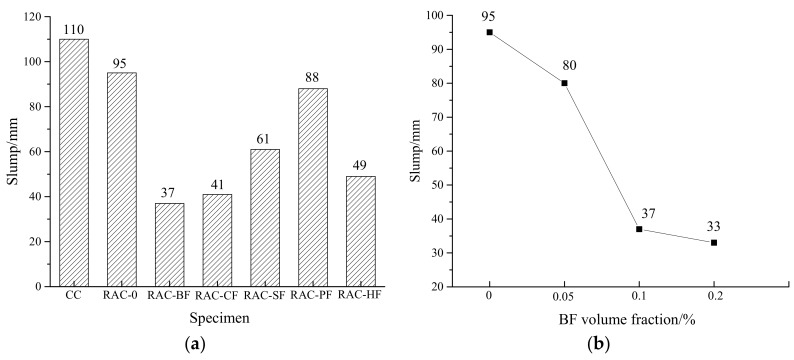
Changes in RAC slumps. (**a**) Different fiber-reinforced RAC; (**b**) Different BF-reinforced RAC.

The workability index of RAC, slumps, are shown in Figure 2. Figure 2a shows that the slump of RAC-0 was lower than that of CC due to the fact that recycled coarse aggregates have higher water absorption reducing the amount of free water. Meanwhile, the slumps of fiber-reinforced RAC were all lower than that of RAC-0 in which RAC-BF and RAC-CF had only 37 mm and 41 mm. This phenomenon lies in the fact that fibers have specific surface areas that should be wrapped by cement mortar. Meanwhile, fibers are easy to cluster during the agitating process, which locks more cement mortar. Moreover, fibers themselves will absorb free water, leading to the slump reduction. The situation became worse for BFs and CFs, as they have a higher water absorption ability than PFs and SFs.

Figure 2b demonstrates that for the BF-reinforced RAC, the slump sharply declined as the volume fraction increased. The slump decreased from 95 mm to 33 mm with the fraction range of 0–0.2%, implying that BF-reinforced RAC requires more water for workability in real-world applications.

### 3.2. Compressive Strengths

**Figure 3:**
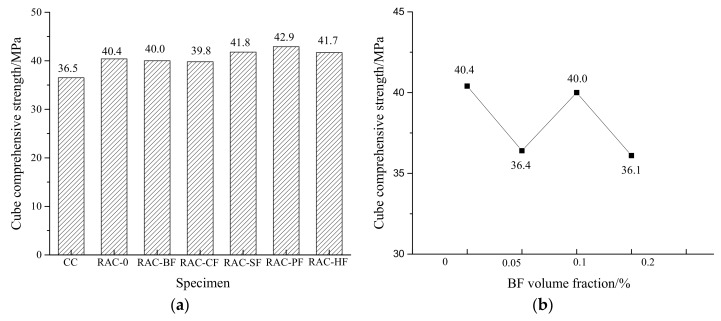
Changes in cube compressive strengths of RAC. (**a**) Different fiber-reinforced RAC; (**b**) Different BF-reinforced RAC.

**Figure 4:**
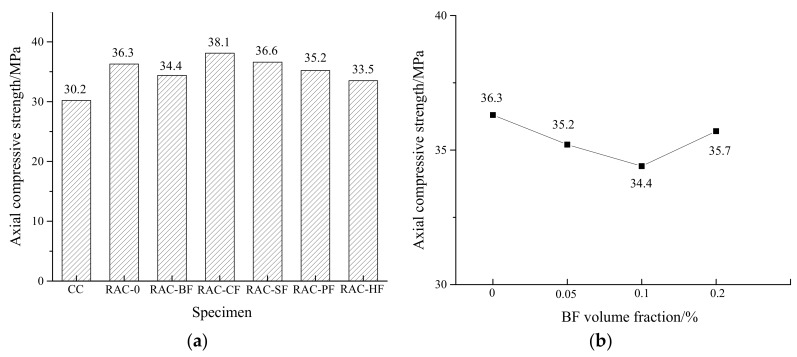
Changes in axial compressive strengths of RAC. (**a**) Different fiber-reinforced RAC; (**b**) Different BF-reinforced RAC.

**Figure 5:**
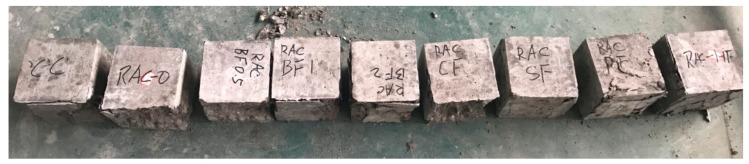
Failure modes of cube compressive specimens.

**Figure 6:**
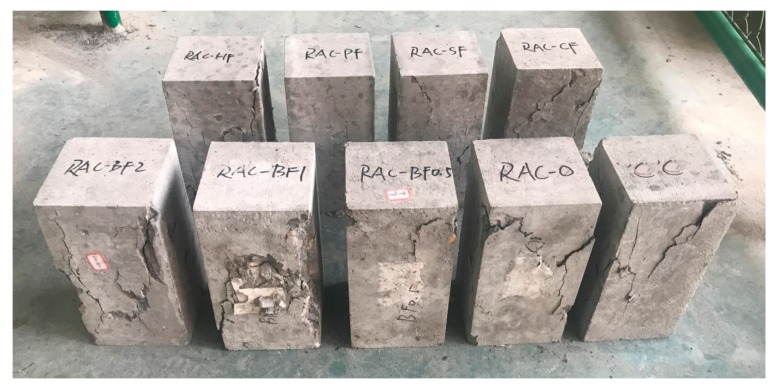
Failure modes of axial compressive specimens.

The cube and axial compressive strengths are shown in Figure 3 and Figure 4. The failure modes of different kinds of specimens are illustrated in Figure 5 and Figure 6.

Figure 3a and Figure 4a show that the compressive and axial strengths of RAC-0 were higher than those of CC, which might be caused by a lower water-cement ratio of RAC. The recycled coarse aggregates absorbed some water resulting in less free water for hydration. However, the compressive strengths of all the fiber-reinforced RAC were similar to that of RAC-0, implying that the fibers did not present much contribution to the compressive strengths under an identical volume fraction of 0.1%. The cube compressive strengths of RAC-PF, RAC-SF and RAC-HF increased by 6.2%, 3.5% and 3.2% respectively, while those of RAC-BF and RAC-CF decreased by 1.0% and 1.5%. As for the axial compression strengths, RAC-CF and RAC-SF increased by 5.0% and 0.8% while the other three decreased by 3.0%, 5.2% and 7.7% respectively. These observations indicate no regularity since it seems different types of fibers showed different effects on the compressive strengths of RAC. It is noted that the slight compressive strength variations of e.g., 1.0% and 0.8% could partly come from the random errors in the tests.

In Figure 3b and Figure 4b, the cube compression strengths of the BF-reinforced RAC show no regularity while the axial compression strengths present a tendency to decline before rising. These observations are similar to those found by Cheng and Li [31] and Liu et al. [32]. Compared to RAC-0, all the BF-reinforced RAC in our tests possessed decreased compression strengths. With respect to the BF volume fractions of 0.05%, 0.1% and 0.2%, the cube compressive strengths decreased by 9.9%, 1.0% and 10.6% respectively. The axial compressive strengths decreased by 3.0%, 5.2% and 1.7%, respectively. The optimal BF fractions were 0.1% and 0.2% for the cube and axial compression strengths, respectively.

The reduction of the compressive strengths of fiber-reinforced RAC might lie in two reasons: (a) the strength reinforcement mechanism of fibers depends on their random and uniform distributions inside concrete, which forms a three-dimensional space truss structure restraining the cracking of concrete. However in reality, it’s very difficult to achieve uniform distributions because fibers are easy to cluster during the agitating process. The clustered fibers produce more defects inside concrete, reducing the concrete strength. (b) fibers will be wrapped by cement mortar during the agitating process. Therefore the effective mortar for concrete itself reduces, leading to lower compressive strengths.

The failure modes of the compressive specimens are shown in Figure 5 and Figure 6. For cube compressive specimens, it can be seen that the failure mode of RAC-0 was similar to that of common concrete. The vertical cracks firstly appeared at the middle height of the specimens and then extended to the corners. At the beginning, the cracks concentrated on the surface of concrete. With the increase of the compressive load, new cracks occurred and gradually expanded to the interior regions with the phenomenon of concrete spalling. However, the failure modes of most fiber-reinforced RAC specimens were relatively intact, implying that the fibers contributed to restraining the crack growth. For the axial compressive specimens, the cracks firstly appeared at the middle heights of the specimens and paralleled to the load direction. Then they extended to the corners with an angle of around 60° to the horizontal until the specimens failed.

### 3.3. Splitting Tensile Strengths

**Figure 7:**
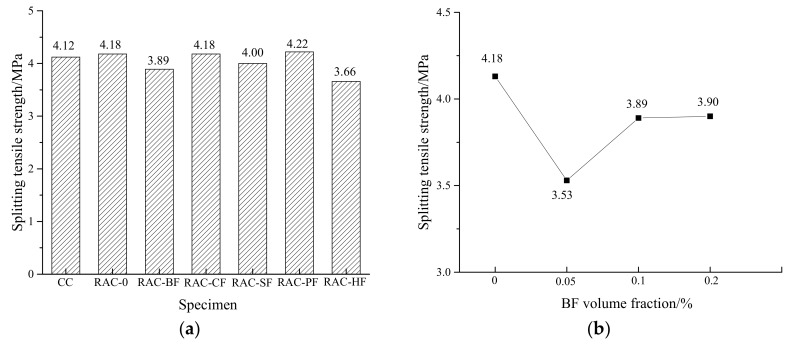
Changes in splitting tensile strengths of RAC. (**a**) Different fiber-reinforced RAC; (**b**) Different BF-reinforced RAC.

The measured splitting tensile strengths are shown in Figure 7.

Figure 7a shows that with the exception of RAC-BF and RAC-HF, the splitting tensile strengths of the RAC specimens were similar to that of CC. The slight difference could be caused by the test random errors. Compared to RAC-0, the strengths of RAC-SF, RAC-BF and RAC-HF decreased by 4.3%, 6.9% and 12.3%, respectively.

Figure 7b presents a fluctuation tendency of the splitting tensile strengths for the BF-reinforced RAC. The strength first declined and then increased as the BF volume fraction increased. With respect to the volume fractions of 0.05%, 0.1% and 0.2%, the strength decreased by 15.5%, 6.9% and 6.7% respectively. This observation was also found by Dong et al. [33] and Li et al. [34].

In addition to the aforementioned factors that affect the compressive strengths, the decrease of the splitting tensile strengths was also due to the inadequate performance of the fibers’ tensile strengths. The fibers embedded in the concrete required an effective anchorage length for full performance. However during the agitating process, BFs and CFs were easy to fracture due to the friction and collision between the aggregates. Thus the actual lengths of some fibers shortened, resulting in the loss of the anchorage lengths. The fractured fibers might induce weak interfaces similar to the initial cracks. On the other hand, the bonding forces between the concrete and fibers might not be strong enough for BFs and CFs. The superplasticizer could also provide certain side effects for the bonding forces.

**Figure 8:**
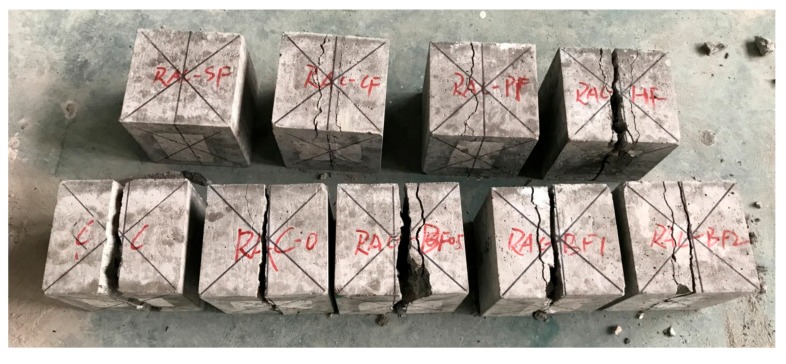
Failure modes of splitting tensile specimens.

The failure modes of the splitting tensile specimens are shown in Figure 8. It can be seen that the failure mode of RAC-0 were similar to that of CC. A major crack appeared at the loading section and split the specimens into two pieces. Regarding to the BF-reinforced specimens, a secondary crack occurred nearby the major one, but didn’t develop throughout the entire cross section. For RAC-SF, RAC-CF and RAC-PF, there were only secondary cracks instead of the major ones. And the specimens were not split into two pieces. These observations indicate that the fibers changed the stress distributions inside the concrete and limited the development of the cracks. Among all the fibers, SFs seemed to provide best performance in restraining the crack development.

### 3.4. Elastic Modulus

**Figure 9:**
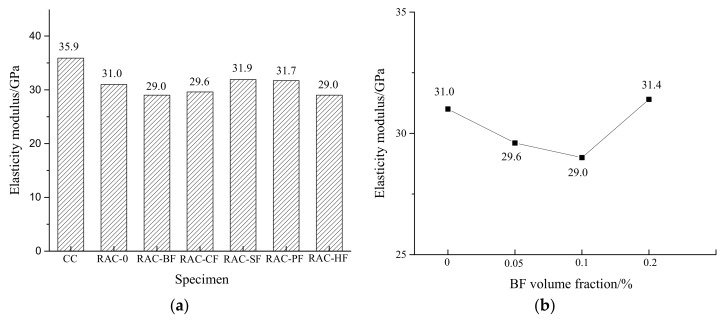
Changes in elasticity modulus of RAC. (**a**) Different fiber-reinforced RAC; (**b**) Different BF-reinforced RAC.

Figure 9a illustrates that the elastic modulus of CC was greater than those of all the RAC, which was caused by the physical defects of the recycled coarse aggregates (e.g., microcracks of aggregates after crushing). Meanwhile, the old mortar adhered to the coarse aggregates also affected the bonding strengths between aggregates and new mortar. These factors led to the decrease of the RAC elastic modulus. Compared to RAC-0, the addition of the fibers had little effect on the elastic modulus of RAC. The elastic modulus of SF- and PF-reinforced specimens increased by 2.9% and 2.3%, while RAC-CF, RAC-BF and RAC-HF decreased by 4.5%, 6.5% and 6.5% respectively.

Figure 9b shows the elastic modulus of the BF-reinforced RAC presented a tendency to decline before rising, which was similar to that of the axial compressive specimens. When the BF volume fraction was 0.05% and 0.1%, the elastic modulus decreased by 4.5% and 6.5% respectively. Then with an increased fraction up to 0.2%, the elastic modulus was greater than that of RAC-0. Nevertheless, the slight difference could be induced by the test random errors.

It is found that the effect of the fibers on the RAC elastic modulus was not obvious or even negative. It is due to the fact that the elastic modulus was measured within the elastic deformation period of the specimens, when cracks hadn’t formed yet and the fibers could not take effect. Moreover, the existence and non-uniform distributions of the fibers created some weak interfaces inside the concrete, inducing a side effect on the elastic modulus of RAC.

## 4. Strength and Elastic Modulus Predictions by BPNNs

**Figure 10:**
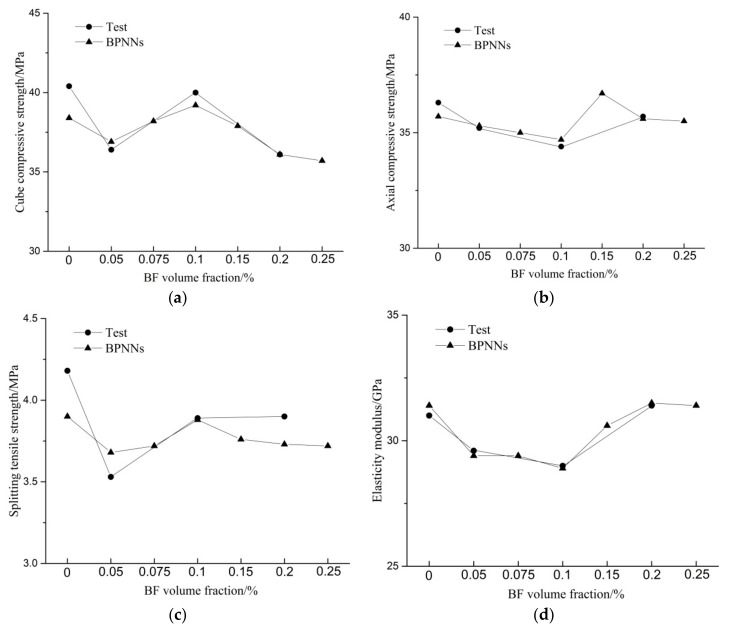
Comparison between BPNNs predictions and test results. (**a**) Cube compressive strengths; (**b**) Axial compressive strengths; (**c**) Splitting tensile strengths; (**d**) Elasticity modulus.

In the interest of verifying the optimal volume fractions of BFs, BPNNs having one hidden layers with 4 neurons were trained using the above test data. Both input and output layers had only one neuron corresponding to the BF volume fractions and the strengths (or the elastic modulus). Table 7 and Figure 10 present the comparison of strengths and elastic modulus between the tests and BPNN predictions. The maximum errors of $f_{\mathrm{cu}}^{\prime }$, $f_{c}^{\prime }$, $f_{\mathrm{sp}}^{\prime }$ and $E_{\mathrm{bf}}^{\prime }$ are 4.9%, 1.6%, 6.7% and 1.3% respectively, proving the satisfactory performance of the BPNNs. It is found that the optimal volume fractions of BF to cube, axial, splitting tensile strengths and the elastic modulus of RAC are 0.1%, 0.15%, 0.1% and 0.2% respectively. Meanwhile, from the prediction curves it seems that the strengths and the elastic modulus became relatively stable when the BF volume fraction was greater than 0.2%.

## 5. Strength Formulas of BF-Reinforced RAC

Based on the above test results, the relationships between the cube compressive strengths and the other three mechanical properties of the BF-reinforced RAC (the axial compressive strengths, the splitting tensile strengths and the elastic modulus) were investigated for the corresponding conversion formulas. Meanwhile, general strength and elastic modulus formulas for the BF-reinforced RAC were also deducted, which could be used for fast estimation of different RAC mechanical properties.

### 5.1. Axial vs. Cube Compressive Strengths

The conversion formula for axial to cube compressive strengths of CC are defined as follows:

(1) $$f_{c}=\alpha_{c}f_{\mathrm{cu}}$$

in which $f_{c}$ and $f_{\mathrm{cu}}$ denote the axial and cube compressive strengths; the conversion factor $\alpha_{c}$ equals to 0.76 and 0.85 according to the Chinese standard (GB50010-2010) [35] and the American design code (ACI 318-11) [36], respectively. This formula was also adopted here for the BF-reinforced RAC, whose test results estimated the conversion factor within the range of 0.86~0.98. Therefore, a median value of 0.92 was taken with the final expression given as

(2) $$f_{c}^{\prime }=0.92f_{\mathrm{cu}}^{\prime }$$

### 5.2. Splitting Tensile vs. Cube Compressive Strengths

The conversion formula for splitting tensile to cube compressive strengths of CC are defined as follows:

(3) $$f_{\mathrm{sp}}=\alpha_{\mathrm{sp}}{\left(f_{\mathrm{cu}}\right)}^{\beta_{\mathrm{sp}}}$$

in which the coefficients $\alpha_{\mathrm{st}}$ and $\beta_{\mathrm{st}}$ equal to 0.19/0.75 and 0.49/0.5 according to the Chinese standard (GB50010-2010) and the American design code (ACI 318-11), respectively. Here the same formula format was adopted for the regression analysis based on the test results.

(4) $$f_{\mathrm{sp}}^{\prime }=0.21{\left(f_{\mathrm{cu}}^{\prime }\right)}^{0.8}$$

The correlation coefficient for the regression is 0.483.

### 5.3. Elastic Modulus vs. Cube Compressive Strengths

The conversion formula for elastic modulus $E_{c}$ to cube compressive strengths of CC are defined as follows:

(5) $$E_{c}=\frac{10^{5}}{2.2+\frac{34.7}{f_{\mathrm{cu}}}}$$

(6) $$E_{c}=4700{\left(f_{\mathrm{cu}}\right)}^{0.5}$$

The two formulas are given by the Chinese standard (GB50010-2010) and the American design code (ACI 318-11), respectively. The former one was taken and modified based on the test results of the BF-reinforced RAC specimens.

(7) $$E_{c}^{\prime }=\frac{10^{5}}{2.3+\frac{35}{f_{\mathrm{cu}}^{\prime }}}$$

### 5.4. Strength Formulas

A general formula for predicting cube compressive, axial compressive and splitting tensile strengths of BF-reinforced RAC is proposed as follows:

(8) $$f_{\mathrm{bf}}^{\prime }=f_{\mathrm{cc}}\left(1+\alpha r+\beta \frac{l}{d}\right)$$

where $f_{\mathrm{bf}}^{\prime }$ denotes the calculated strength of BF-reinforced RAC; $f_{\mathrm{cc}}$ denotes the strength of common concrete, which can be either one of $f_{\mathrm{cu}}$, $f_{c}$ and $f_{\mathrm{sp}}$; *r* is the substitution rate of recycled aggregates; α is a factor embodying the influence of the water absorption of recycled aggregates on the water-cement ratio; β is the coefficient for the BF volume fraction of different types of specimens and it equals to 0 for RAC with no fibers; *l* and *d* represent the length and diameter of BFs. By fitting the test results, the coefficients *α* and *β* were obtained and listed in Table 8.

A recommended formula for predicting the elastic modulus of BF-reinforced RAC is also proposed as follows

(9) $$E_{\mathrm{bf}}^{\prime }=E_{\mathrm{cc}}\cdot \left(1+\eta r+\beta_{E}\frac{l}{d}\right)$$

where $E_{\mathrm{bf}}^{\prime }$ denotes the calculated elastic modulus of BF-reinforced RAC; $E_{\mathrm{cc}}$ denotes is the elastic modulus of common concrete; η represents the elastic modulus reduction factor of RAC, which is determined as −0.27 by regressing the test data; $\beta_{E}$ is the coefficient the BF volume fraction estimated by fitting the experimental results

(10) $$\beta_{E}=\left(0.9-110\rho +550\rho^{2}\right)\times 10^{-5}$$

Lastly, the predicted and measured strengths ($S_{p}$ and $S_{m}$), together with the elastic modulus, were compared and listed in Table 9. It can be seen that the predictions agree well with the test measurements, indicating the satisfactory performance of the proposed formulas.

## 6. Conclusions

The mechanical properties of fiber-reinforced RAC with different types of fibers and various BF volume fractions have been experimentally studied. The cube compressive, axial compressive and splitting tensile strengths, together with the elastic modulus, of different fiber-reinforced RAC were compared with a further investigation on the variation of BF volume fractions. Based on the test results, the conversion and strength formulas of BF-reinforced RAC were also deducted, which is subsequently verified by the BPNNs to find the optimal BF volume fraction corresponding to each mechanical property. Some conclusions are drawn as below:

(1) In certain cases, the compressive and splitting tensile strengths of RAC could be greater than those of CC under identical mixture ratios due to the water absorption of recycled coarse aggregates.
(2) The addition of fibers can induce positive or negative effects on the mechanical properties of RAC. The latter effect could be caused by the non-uniform distributions of fibers inside concrete. However, different fibers show different contributions to the failure modes of RAC.
(3) Based on the test results, the optimal BF volume fractions for cube compressive, axial compressive, splitting tensile strength and elastic modulus of RAC were 0.1%, 0.2%, 0.2% and 0.2% respectively. Moreover, the optimal fractions were refined by using the BPNNs as 0.1%, 0.15%, 0.1% and 0.2% respectively.
(4) The conversion formulas between cube compressive strength and other mechanical properties can be established on the design codes. The predictions by the proposed strength and elastic modulus formulas agree well with the test measurements.

## Figures and Tables

**Table 1:** Physical properties of coarse aggregates.

Aggregates	Grading (mm)	Bulk Density (kg/m^3^)	Apparent Density (kg/m^3^)	Water Absorption (%)	Crush Index (%)
NCA	5–20	1372	2790	0.4	5.1
RCA	5–20	1215	2491	3.4	10.0

**Table 2:** Physical and mechanical properties of fibers.

Fiber	Equivalent Diameter (mm)	Length (mm)	Density (g/cm^3^)	Tensile Strength (MPa)	Elastic Modulus (GPa)
BF	0.015	18	2.65	2630	88.9
CF	0.085	9	1.76	3700	230
PF	0.0182	19	0.91	556	8.9
SF	0.33	37	7.8	658	220

**Table 3:** Mix proportion of different fiber-reinforced RAC (kg/m^3^).

Specimen	Cement	Sand	NA	RA	Fly Ash	Plasticizer	Water	Fiber
CC	337	648	1152	0	37.4	2.2	205	0
RAC-0	337	648	576	576	37.4	2.2	205	0
RAC-BF	337	648	576	576	37.4	2.2	205	2.65
RAC-CF	337	648	576	576	37.4	2.2	205	2.4
RAC-SF	337	648	576	576	37.4	2.2	205	7.8
RAC-PF	337	648	576	576	37.4	2.2	205	0.9
RAC-HF	337	648	576	576	37.4	2.2	205	0.45 + 1.3

Note: RAC-0 and RAC-BF refer to RAC without fibers and strengthened by basalt fibers, respectively.

**Table 4:** Mix proportion of BF-reinforced RAC (kg/m^3^).

Specimen	Cement	Sand	NA	RA	Fly Ash	Plasticizer	Water	Fiber
RAC-0	337	648	576	576	37.4	2.2	205	0
RAC-BF0.05	337	648	576	576	37.4	2.2	205	1.32
RAC-BF0.1	337	648	576	576	37.4	2.2	205	2.65
RAC-BF0.2	337	648	576	576	37.4	2.2	205	5.26

Note: RAC-BF0.05 refers to basalt fiber reinforced RAC with a volume fraction of 0.05%.

**Table 5:** Test results of different fiber-reinforced RAC specimens.

Specimen	Slump (mm)	*f*_cu_ (MPa)	*f*_c_ (MPa)	*f*_sp_ (MPa)	*E* (MPa)
CC	110	36.5	30.2	4.12	35.9
RAC-0	95	40.4	36.3	4.18	31.0
RAC-BF	37	40.0	34.4	3.89	29.0
RAC-CF	41	39.8	38.1	4.18	29.6
RAC-SF	61	41.8	36.6	4.00	31.9
RAC-PF	88	42.9	35.2	4.22	31.7
RAC-HF	49	41.7	33.5	3.66	29.0

**Table 6:** Test results of RAC specimens having different BF volume fractions.

Specimen	Slump (mm)	*f*_cu_ (MPa)	*f*_c_ (MPa)	*f*_sp_ (MPa)	*E* (MPa)
RAC-0	95	40.4	36.3	4.18	31.0
RAC-BF0.05	80	36.4	35.2	3.53	29.6
RAC-BF0.1	37	40.0	34.4	3.89	29.0
RAC-BF0.2	33	36.1	35.7	3.90	31.4

**Table 7:** Comparison between tests and BPNN predictions.

BF Volume Fraction (%)		0	0.05	0.075	0.1	0.15	0.2	0.25
$f_{\mathrm{cu}}^{\prime }$ (MPa)	test	40.4	36.4	-	40	-	36.1	-
	BPNNs	38.4	36.9	38.2	39.2	37.9	36.1	35.7
$f_{c}^{\prime }$ (MPa)	test	36.3	35.2	-	34.4	-	35.7	-
	BPNNs	35.7	35.3	35	34.7	36.7	35.6	35.5
$f_{\mathrm{sp}}^{\prime }$ (MPa)	test	4.18	3.53	-	3.89	-	3.9	-
	BPNNs	3.9	3.68	3.72	3.88	3.76	3.73	3.72
$E_{\mathrm{bf}}^{\prime }$ (MPa)	test	31	29.6	-	29	-	31.4	-
	BPNNs	31.4	29.4	29.4	28.9	30.6	31.5	31.4

**Table 8:** *α* and *β* in the strength formula.

$f_{\mathrm{bf}}^{\prime }$	*α*	*β*
$f_{\mathrm{cu}}^{\prime }$	0.21	(−2.6 + 42*ρ* − 170*ρ*^2^) × 10^−4^
$f_{c}^{\prime }$	0.40	(0.2 − 12.4*ρ* + 53*ρ*^2^) × 10^−4^
$f_{\mathrm{sp}}^{\prime }$	0.03	(−2.5 + 29*ρ* − 96*ρ*^2^) × 10^−4^

Note: *ρ* refers to the volume fraction of BFs.

**Table 9:** Comparison between strength formula predictions and test results.

Type	Fiber Volume Fraction (%)	*S*_p_	*S*_m_	*S*_p_/*S*_m_	Type	Fiber Volume Fraction (%)	*S*_p_	*S*_m_	*S*_p_/*S*_m_
$f_{\mathrm{cu}}^{\prime }$	0	40.3	40.4	0.998	$f_{\mathrm{sp}}^{\prime }$	0	4.18	4.18	1.000
	0.05	36.3	36.4	0.997		0.05	3.54	3.53	1.004
	0.1	39.9	40.0	0.997		0.1	3.90	3.89	1.004
	0.2	36.0	36.1	0.996		0.2	3.91	3.90	1.004
$f_{c}^{\prime }$	0	36.2	36.3	0.998	$E_{\mathrm{bf}}^{\prime }$	0	31.1	31.0	1.002
	0.05	35.2	35.2	1.000		0.05	29.7	29.6	1.002
	0.1	34.4	34.4	1.000		0.1	29.1	29.0	1.002
	0.2	35.7	35.7	0.999		0.2	31.4	31.4	1.001

## References

[1] Dong J.F., Wang Q.Y., Guan Z.W. (2013). Structural behaviour of recycled aggregate concrete filled steel tube columns strengthened by CFRP. Eng. Struct..

[2] Colangelo F., Forcina A., Farina I., Petrillo A. (2018). Life Cycle Assessment (LCA) of Different Kinds of Concrete Containing Waste for Sustainable Construction. Buildings.

[3] Colangelo F., Petrillo A., Cioffi R., Borrelli C., Forcina A. (2018). Life cycle assessment of recycled concretes: A case study in southern Italy. Sci. Total Environ..

[4] Silva R.V., Brito J.D., Dhir R.K. (2014). Properties and composition of recycled aggregates from construction and demolition waste suitable for concrete production. Constr. Build. Mater..

[5] Hu J., Wang Z., Kim Y. (2013). Feasibility study of using fine recycled concrete aggregate in producing self-consolidation concrete. J. Sustain. Cem.-Based Mater..

[6] Xiao J.Z., Lu D., Ying J.W. (2013). Durability of Recycled Aggregate Concrete: An Overview. J. Adv. Concr. Technol..

[7] Lawania K., Sarker P., Biswas W. (2015). Global Warming Implications of the Use of By-Products and Recycled Materials in Western Australia’s Housing Sector. Materials.

[8] Dong H., Cao W., Bian J., Zhang J. (2014). The Fire Resistance Performance of Recycled Aggregate Concrete Columns with Different Concrete Compressive Strengths. Materials.

[9] Souche J.C., Devillers P., Salgues M., Diaz E.G. (2017). Influence of recycled coarse aggregates on permeability of fresh concrete. Cem. Concr. Compos..

[10] Evangelista L., Brito J.D. (2014). Concrete with fine recycled aggregates: A review. Eur. J. Environ. Civ. Eng..

[11] Colangelo F., Cioffi R. (2017). Mechanical properties and durability of mortar containing fine fraction of demolition wastes produced by selective demolition in South Italy. Compos. Part B.

[12] Etxeberria M., Vázquez E., Marí A., Barra M. (2007). Influence of amount of recycled coarse aggregates and production process on properties of recycled aggregate concrete. Cem. Concr. Res..

[13] Huda S.B., Alam M.S. (2015). Mechanical and freeze-thaw durability properties of recycled aggregate concrete made with recycled coarse aggregate. J. Mater. Civ. Eng..

[14] Liu W.C., Cao W.L., Zhang J.W., Wang R.W., Ren L.L. (2017). Mechanical Behavior of Recycled Aggregate Concrete-Filled Steel Tubular Columns before and after Fire. Materials.

[15] Marie I., Quiasrawi H. (2012). Closed-loop recycling of recycled concrete aggregates. J. Clean. Prod..

[16] Wagih A.M., El-Karmoty H.Z., Ebid M., Okba S.H. (2013). Recycled construction and demolition concrete waste as aggregate for structural concrete. HBRC J..

[17] Gayarre F.L., Pérez L.C., López M.A.S., Cabo A.D. (2014). The effect of curing conditions on the compressive strength of recycled aggregate concrete. Constr. Build. Mater..

[18] Evangelista L., Brito J. (2007). Mechanical behaviour of concrete made with fine recycled concrete aggregates. Cem. Concr. Compos..

[19] Nixon P.J. (1978). Recycled concrete as an aggregate for concrete—A review. Mater. Constr..

[20] Khatib J.M. (2005). Properties of concrete incorporating fine recycled aggregate. Cem. Concr. Res..

[21] Loo Y.H., Tam C.T., Ravindrarajah R.S. (1987). Recycled concrete as fine and coarse aggregate in concrete. Mag. Concr. Res..

[22] Lu X.B., Hsu C.T.T. (2006). Behavior of high strength concrete with and without steel fiber reinforcement in triaxial compression. Cem. Concr. Res..

[23] Asokan P., Osmani M., Price A.D.F. (2009). Assessing the recycling potential of glass fibre reinforced plastic waste in concrete and cement composites. J. Clean. Prod..

[24] Lee S.C., Oh J.H., Cho J.Y. (2015). Compressive Behavior of Fiber-Reinforced Concrete with End-Hooked Steel Fibers. Materials.

[25] Kim S.B., Yi N.H., Kim H.Y., Kim J.H.J., Song Y.C. (2010). Material and structural performance evaluation of recycled PET fiber reinforced concrete. Cem. Concr. Compos..

[26] Jiang C.H., Fan K., Wu F., Chen D. (2014). Experimental study on the mechanical properties and microstructure of chopped basalt fibre reinforced concrete. Mater. Des..

[27] Ayub T., Shafiq N., Nuruddin M.F. (2014). Effect of Chopped Basalt Fibers on the Mechanical Properties and Microstructure of High Performance Fiber Reinforced Concrete. Adv. Mater. Sci. Eng..

[28] Alnahhal W., Aljidda O. (2018). Flexural behavior of basalt fiber reinforced concrete beams with recycled concrete coarse aggregates. Constr. Build. Mater..

[29] Bishop C.M. (1995). Neural Networks for Pattern Recognition.

[30] GB/T 50081-2002 (2002). Standard for Test Method of Mechanical Properties on Ordinary Concrete.

[31] Cheng T.H., Li Y.X. (2017). Experimental study on mechanical properties of basalt fiber reinforced concrete. Chin. Concr. Cem. Prod..

[32] Liu H.X., Yang J.W., Kong X.Q., Xue X.X. (2017). Basic Mechanical Properties of Basalt Fiber Reinforced Recycled Aggregate Concrete. Open Civ. Eng. J..

[33] Dong J.F., Wang Q.Y., Guan Z.W. (2017). Material properties of basalt fibre reinforced concrete made with recycled earthquake waste. Constr. Build. Mater..

[34] Li X.L., Jin B.H., Yao Y.F., Guo H.W. (2017). Mechanical properties of basalt fiber reinforced recycled aggregate concrete. J. Hebei Uni. (Nat. Sci. Ed.).

[35] GB 50010-2010 (2010). Code for Design of Concrete Structures.

[36] ACI 318-11 (2011). Building Code Requirements for Structural Concrete and Commentary.

